# Supplementary material for: Lightweight mobile network for real-time violence recognition
Source: PLoS One. 2022 Oct 31;17(10):e0276939. doi: 10.1371/journal.pone.0276939 (PMC9621415; doi:10.1371/journal.pone.0276939)
Supplement: Attachment — Submitted filename: Response to Reviewers.docx [file pone.0276939.s001.docx]

Dear editors and reviewers,

Thank you very much for your nice consideration of our manuscript and kind help with the reviewing of the manuscript entitled “Lightweight mobile network for real-time violence recognition” (Manuscript Number: PONE-D-22-21672 ). Also, we highly appreciate that the reviewers and editors gave some constructive and objective comments to this manuscript. Those comments are very helpful for revising and improving our manuscript. Here we submit a new version of the manuscript, which has been carefully revised according to comments of the reviewers and editors. All changes are highlighted in blue in the revised manuscript. Below is our detailed response to all of the reviewer’s comments.

**Comments of ACADEMIC EDITOR:**

The research proposes a MobileNet-TSM, a lightweight network for real time voice recognition. It has an acceptable structure and the experiments seems enough to show the performance. However, there are some comments to be considered before possible publication.
1-Explanation and justification for the use of MobileNet V2 as the backbone structure to be provided.
2-The abstract need to be improved, more about the advantage and disadvantages of your approach, novelty, and valuable results you observed.
3-For literature review, further discussions on these hot issues, you should supplement some newest references to papers published more recently, which appeared from 2021 on.
4-Every equation should be explained more clearly.
5-Table 4 and Table 5 results should be interpreted in detail.

**Response:**

Thank you very much for your previous comments. Those comments had helped us a lot for improving our manuscript.

**Comment 1:**

Explanation and justification for the use of MobileNet V2 as the backbone structure to be provided.

**Response:**

Thank you for your advice. In line 140-163, more explanation has already been added to explain why we ended up using MobileNet V2 as the backbone structure.

**Comment 2:**

The abstract need to be improved, more about the advantage and disadvantages of your approach, novelty, and valuable results you observed.

**Response:**

Thank you for your advice. We rewrite the abstract to show the advantages and disadvantages of our proposed method. The design purpose and performance metrics are stated in detail to show the valuable results we have observed.

**Comment 3:**

For literature review, further discussions on these hot issues, you should supplement some newest references to papers published more recently, which appeared from 2021 on.

**Response:**

Thank you for your advice. In line 47-67, seven newest references on violence recognition has already been added. One of them (line 131-138) is our recent research results, which is also the basis of this manuscript. We also compare them in Table 4 and Table 5.

**Comment 4:**

Every equation should be explained more clearly.

**Response:**

Thank you for your advice. In line 121-130, we have explained each equation and added some easy-to-understand derivations.

**Comment 5:**

Table 4 and Table 5 results should be interpreted in detail.

**Response:**

Thank you for your advice. In line 272-287 and 306-312, we illustrate the results presented in Tables 4 and Table 5 in detail and compare with state-of-the-art algorithms.

**Comments of Reviewer 1:**

1. The objectives need to be well stated in the abstract
2. Performance metrics were not stated
3. The summary of the results should be stated in the abstract. Extensive experiments were conducted on three public datasets (Hockey Fights, Crowd Violence, and RWF-2000) to prove the validity of this method. What are the results of the experiment?
4. At least two more Keywords should be added
5. What is the trade-off of this proposed method?
6. What is the difference between this paper and A Lightweight Network for Violence Detection, ICIGP 2022: 2022 the 5th International Conference on Image and Graphics Processing (ICIGP)January 2022 Pages 15–21 [https://doi.org/10.1145/3512388.3512391](https://doi.org/10.1145/3512388.3512391) ?
7. What informed the consideration of the deep separable convolution as the initial bottleneck.
8. The research gap in this study was not well established.

**Response:**

Thank you very much for your previous comments. Those comments had helped us a lot for improving our manuscript.

**Comment 1,2 and 3:**

The objectives need to be well stated in the abstract.

Performance metrics were not stated.

The summary of the results should be stated in the abstract. Extensive experiments were conducted on three public datasets (Hockey Fights, Crowd Violence, and RWF-2000) to prove the validity of this method. What are the results of the experiment?

**Response:**

Thank you for your advice. We rewrite the abstract to show the advantages and disadvantages of our proposed method. The design purpose and performance metrics are stated in detail to show the valuable results we have observed.

**Comment 4:**

At least two more Keywords should be added.

**Response:**

Thank you for your advice. We have added three keywords after the abstract.

**Comment 5 and 8:**

What is the trade-off of this proposed method?

The research gap in this study was not well established.

**Response:**

Thank you for your advice. In line 272-287 and 306-312, we illustrate the results presented in Tables 4 and Table 5 in detail and compare with state-of-the-art algorithms to show the improvement of our proposed method.

In short, this method greatly reduced the model size, at the cost of an accuracy gap of about 3% compared with the existing methods. At the same time, we also built an Android terminal violence recognition application.

**Comment 6:**

What is the difference between this paper and A Lightweight Network for Violence Detection, ICIGP 2022: 2022 the 5th International Conference on Image and Graphics Processing (ICIGP)January 2022 Pages 15–21 [https://doi.org/10.1145/3512388.3512391](https://doi.org/10.1145/3512388.3512391) ?

**Response:**

Thank you for your advice. In line 279-287, we explained the similarities and differences between the methods proposed in this manuscript and A Lightweight Network for Violence Detection.

**Comment 7:**

What informed the consideration of the deep separable convolution as the initial bottleneck.

**Response:**

Thank you for your advice. In line 140-163, more explanation has already been added to explain why we ended up using MobileNet V2 as the backbone structure. The depthwise separable convolution is the main innovation of MobileNet V2, which can greatly reduce the complexity of the model while maintaining reliable accuracy.

**Comments of Reviewer 2:**

Introduction can be reframed including the objectives of the article.
Literature survey is not adequate. Many new papers on machine learning/deep learning algorithms are not included.
proposed method(MobileNet-TSM) is not explained well. Methodology is not adequate.
chosen Parameters are very low for analyzing.
Incorporate the comparison graph.

**Response:**

Thank you very much for your previous comments. Those comments had helped us a lot for improving our manuscript.

**Comment 1 and 2:**

Introduction can be reframed including the objectives of the article.

**Response:**

Thank you for your advice. In line 68-73, we have reframed the introduction to include some newest researches and separately state our research objectives.

**Comment 2:**

Literature survey is not adequate. Many new papers on machine learning/deep learning algorithms are not included.

**Response:**

Thank you for your advice. In line 47-67, seven newest references on violence recognition has already been added. One of them (line 131-138) is our recent research results, which is also the basis of this manuscript. We also compare them in Table 4 and Table 5.

**Comment 3:**

Proposed method(MobileNet-TSM) is not explained well. Methodology is not adequate.

**Response:**

Thank you for your advice. In line 140-163, more explanation has already been added to explain why we ended up using MobileNet V2 as the backbone structure. The depthwise separable convolution is the main innovation of MobileNet V2, which can greatly reduce the complexity of the model while maintaining reliable accuracy.

In Figure 4, we show the improved body structure and explain how to use the temporal shift modules in the paragraph Details.

**Comment 4:**

Chosen Parameters are very low for analyzing.

**Response:**

Thank you for your advice. In line 239-241, more parameter setting details are provided.

**Comment 5:**

Incorporate the comparison graph.

**Response:**

Thank you for your advice. After our discussion, we kept Tables 4 and 5 for the following reasons. First, discussing from the perspective of accuracy and computational cost can more highlight the trade-offs of our proposed model. Second, if the comparison graph is merged, the horizontal distance of the table will be too large.

**Comments of Reviewer 3:**

1. In Page 2, Line 22, Can you supply more explanation to Oriented VIolent Flows (OViF) [6] ?
   2. In Page 3, Line 57, how do you exactly deploy the trained model on the Android terminal ?
   3. In Page 7, Line 222, why do you use torchsummary to calculate models’ parameters ?
   4. In Page 8, Line 242, Why cant you use latest version instead of PyTorch version 1.9.0 ?
   5. Can you give the scope for future enhancement of this research work ?

**Comment 1:**

In Page 2, Line 22, Can you supply more explanation to Oriented VIolent Flows (OViF) [6] ?

**Response:**

Thank you for your advice. In line 25-28, more explanation has already been added.

**Comment 2:**

In Page 3, Line 57, how do you exactly deploy the trained model on the Android terminal ?

**Response:**

Thank you for your advice. In Page 10, line 321-324, more explanation has already been added to describe how we deploy the pretrained model on the Android terminal. Two images show the parts of our code mentioned.

**Comment 3:**

In Page 7, Line 222, why do you use torchsummary to calculate models’ parameters ?

**Response:**

Thank you for your advice. Because torchsummary can control all variables of model computational complexity, and the calculation results can be simply reviewed instead of manual calculation. Params shown in Table 5 are the amount of learnable parameters of our experimental model which are independent of the image input size. Estimated total size is affected by the size of the input image. So to make the data comparable and reproducible, we used torchsummary to calculate models’ parameters. In Page 9, line 297-298, more explanation has already been added.

**Comment 4:**

In Page 8, Line 242, Why cant you use latest version instead of PyTorch version 1.9.0 ?

**Response:**

Thank you for your advice. In fact, we have used PyTorch 1.10.0 and PyTorch 1.11.0. The version problems we encountered while training our models have been resolved. However, in order to deploy the trained model on the Android terminal, PyTorch version must be the same as Pytorch-android (https://pytorch.org/mobile/android/). If this is not done, there will be compatibility problems. We scoured many blogs and forums. But in the end we can only use PyTorch version 1.9.0 to do our work. In Page 10, line 319, more explanation has already been added.

**Comment 5:**

Can you give the scope for future enhancement of this research work ?

**Response:**

Thank you for your advice. We put forward some superficial views in the conclusion of the manuscript, in Page 11, line 344-347.
