# Supplementary material for: Lightweight mobile network for real-time violence recognition
Source: PLoS One. 2022 Oct 31;17(10):e0276939. doi: 10.1371/journal.pone.0276939 (PMC9621415; doi:10.1371/journal.pone.0276939)
Supplement: Attachment — Submitted filename: Response to Reviewers.docx [file pone.0276939.s002.docx]

Dear editors and reviewers,

Thank you very much for your nice consideration of our manuscript and kind help with the reviewing of the manuscript entitled “Lightweight mobile network for real-time violence recognition” (Manuscript Number: PONE-D-22-21672R1 ). Also, we highly appreciate that the reviewers and editors gave some constructive and objective comments to this manuscript. Those comments are very helpful for revising and improving our manuscript. Here we submit a new version of the manuscript, which has been carefully revised according to comments of the reviewers and editors. All changes are highlighted in blue in the revised manuscript. Below is our detailed response to all of the reviewer’s comments.

**Comments of ACADEMIC EDITOR:**

The quality of the images has to be drastically improved for further progress of the article.

**Response:**

Thank you very much for your previous comments. Those comments had helped us a lot for improving our manuscript. All pictures have been redrawn. Preflight Analysis and Conversion Engine (PACE) digital diagnostic tool have been used to ensure figures meet PLOS requirements.

**Comments of Reviewer 1:**

(No Response)

**Response:**

Thank you very much for your previous comments. Those comments had helped us a lot for improving our manuscript.

**Comments of Reviewer 2:**

Image Quality must be enhanced. (At least 300 dpi).

Fig.5 and Fig.6 originality is still not adequate.

Comparison graphs shall be added.
**Response:**

Thank you very much for your previous comments. Those comments had helped us a lot for improving our manuscript.

**Comment 1:**

Image Quality must be enhanced. (At least 300 dpi).

**Response:**

Thank you for your advice. All pictures have been redrawn. Preflight Analysis and Conversion Engine (PACE) digital diagnostic tool have been used to ensure figures meet PLOS requirements.

**Comment 2:**

Fig.5 and Fig.6 originality is still not adequate..

**Response:**

Thank you for your advice. After our team's discussion, we thought that Figure 5 and Figure 6 only played a role of demonstration, so we decided to delete these two pictures.

**Comment 3:**

Comparison graphs shall be added.

**Response:**

Thank you for your advice. Next to Table 4 and Table 5, we selected important performance indicators and made comparison graphs.

**Comments of Reviewer 3:**

All comments have been addressed by the authors.

**Response:**

Thank you very much for your previous comments. Those comments had helped us a lot for improving our manuscript.
